# Apically-located P4-ATPase1-Lem1 complex internalizes phosphatidylserine and regulates motility-dependent invasion and egress in *Toxoplasma gondii*

**DOI:** 10.1016/j.csbj.2023.02.032

**Published:** 2023-02-18

**Authors:** Kai Chen, Xiyu Huang, Ute Distler, Stefan Tenzer, Özlem Günay-Esiyok, Nishith Gupta

**Affiliations:** a Department of Molecular Parasitology, Faculty of Life Sciences, Humboldt University, Berlin, Germany; b Institute of Immunology, University Medical Center of the Johannes-Gutenberg University, Mainz, Germany; c Intracellular Parasite Education and Research Labs (iPEARL), Department of Biological Sciences, Birla Institute of Technology and Science, Pilani (BITS-P), Hyderabad, India

**Keywords:** mAID, (mini) auxin-inducible degron, BSA, bovine serum albumin, COS, crossover sequence, DAPI, 4′,6-diamidino-2-phenylindole, DHFR-TS, dihydrofolate reductase – thymidylate synthase, HFF, human foreskin fibroblast, HXGPRT, hypoxanthine-xanthine-guanine phosphoribosyltransferase, IAA, indole-3-acetic acid, NBD, nitrobenzoxadiazole, PBS, phosphate-buffered saline, PtdCho, phosphatidylcholine, PtdEtn, phosphatidylethanolamine, PtdSer, phosphatidylserine, PtdThr, phosphatidylthreonine, CDC50, Cell Division Control 50, LEM, Ligand Effector Module, cGMP, cyclic Guanosine Monophosphate, UTR, untranslated region, NBD-lipid, Phosphatidylserine, P4-ATPase1, Lem1, Cdc50, Phospholipid flipping

## Abstract

The membrane asymmetry regulated by P4-ATPases is crucial for the functioning of eukaryotic cells. The underlying spatial translocation or flipping of specific lipids is usually assured by respective P4-ATPases coupled to conforming non-catalytic subunits. Our previous work has identified five P4-ATPases (*Tg*P4-ATPase1–5) and three non-catalytic partner proteins (*Tg*Lem1–3) in the intracellular protozoan pathogen, *Toxoplasma gondii*. However, their flipping activity, physiological relevance and functional coupling remain unknown. Herein, we demonstrate that *Tg*P4-ATPase1 and *Tg*Lem1 work together to translocate phosphatidylserine (PtdSer) during the lytic cycle of *T. gondii*. Both proteins localize in the plasma membrane at the invasive (apical) end of its acutely-infectious tachyzoite stage. The genetic knockout of P4-ATPase1 and conditional depletion of Lem1 in tachyzoites severely disrupt the asexual reproduction and translocation of PtdSer across the plasma membrane. Moreover, the phenotypic analysis of individual mutants revealed a requirement of lipid flipping for the motility, egress and invasion of tachyzoites. Not least, the proximity-dependent biotinylation and reciprocal immunoprecipitation assays demonstrated the physical interaction of P4-ATPase1 and Lem1. Our findings disclose the mechanism and significance of PtdSer flipping during the lytic cycle and identify the P4-ATPase1-Lem1 heterocomplex as a potential drug target in *T. gondii*.

## Graphical Abstract

**Figure:**
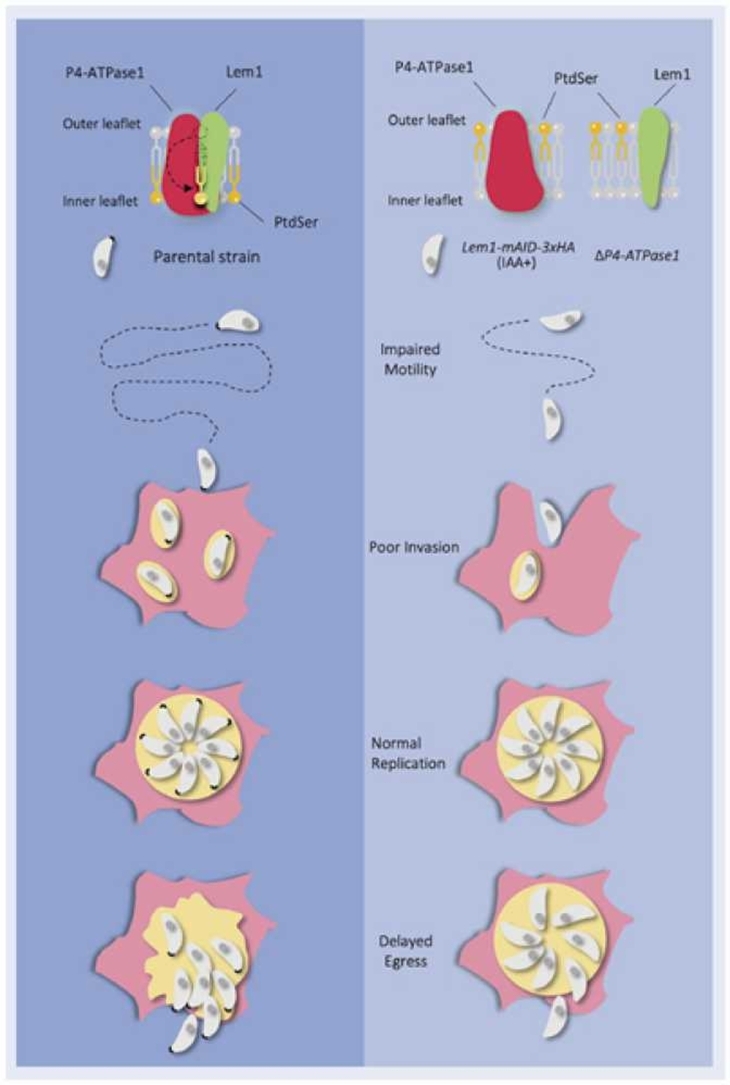

## 1 Introduction

In eukaryotic cells, numerous subcellular pathways depend on the phospholipid composition of the membrane bilayers, which is pivotal to diverse functions, such as maintenance of the surface charge, membrane curvature and permeability, vesicular trafficking (exocytosis, endocytosis) and folding of proteins [1], [2], [3], [4], [5]. Phospholipids, comprising a significant fraction of cellular lipids, are asymmetrically present in the membranes. Phosphatidylcholine (PtdCho) and sphingomyelin are usually positioned on the exofacial leaflet, whereas phosphatidylserine (PtdSer) and phosphatidylethanolamine (PtdEtn) are abundant on the cytosolic side of the plasma membrane in most cell types [2], [5], [6] with a few exceptions. For instance, PtdCho is dominantly located on the cytosolic leaflet of the plasmalemma and Golgi in budding yeast, but it is distributed equally between the cytosolic and luminal leaflets of other organelles. Similarly, the symmetrical distribution of PtdCho has also been seen in some mammalian cells [7]. The ATP-dependent transporters termed P4-ATPases, or flippases control this phospholipid distribution in an energy-dependent manner [8], [9]. Lipid-translocating P4-ATPases (α-subunits) usually couple with a member of the non-catalytic β subunits, which are evolutionarily conserved across eukaryotes [9], [10], [11], [12]. The β subunit proteins present in yeast are termed the Ligand Effector Module 3 (LEM3) or Ro-Sensitive 3 (ROS3), Cell Division Control 50 (CDC50) and transcription factor CRF1, whereas all known mammalian β subunits belong to the CDC50 family [13], [14], [15]. The β-subunits assist in the folding of corresponding α-subunits (P4-ATPase), and ensure stable expression, localization and activation of P4-ATPases for lipid flipping by forming a heteromeric complex [6], [11], [12], [16], [17], [18], [19].

The protozoan phylum Apicomplexa comprises over 6000 obligate intracellular parasite species of animals and humans. *Toxoplasma* and *Plasmodium* are the two exemplary pathogens of this group deployed to decipher the concepts of intracellular parasitism. In the context of this work, phospholipid synthesis and transport have been active areas of research in both parasites [20], [21], [22], [23], [24], [25], [26], [27], [28]; however, their roles in parasite signaling have emerged only recently after discovering exclusive alveolate-specific P4-ATPase-conjugated guanylate cyclase (ATPase_P_-GC) proteins. A series of independent studies collectively link lipid flipping with cGMP signaling and associated lifecycle events in *Toxoplasma* and *Plasmodium* species [29], [30], [31], [32], [33], [34]. Two of these studies focusing on *T. gondii* [29] and *P. yoelii* [33] also disclosed a signaling complex with other interaction partners, including a LEM/CDC50 family protein. The data show the importance of the P4-ATPase domain for cGMP signaling and parasite virulence. More recently, we described five additional P4-ATPases (*Tg*P4-ATPase1–5) and their potential β-subunits (*Tg*Lem1–3) in *T. gondii* [27]. The signatures of typical P4-ATPases, including ten transmembrane helices, and actuator (**A**), nucleotide-binding (**N**) and phosphorylation (**P**) domains, are conserved in *Tg*P4-ATPase1–5. Not least, all except *Tg*P4-ATPase4 are expressed during the lytic cycle of *T. gondii* and display varied subcellular localization in tachyzoites.

*Tg*P4-ATPase1 resides in the plasma membrane at the (invasive) apical end of the tachyzoite stage, while *Tg*P4-ATPase2 and *Tg*P4-ATPase5 are present in the plasmalemma and cytomembranes. *Tg*P4-ATPase3 is expressed in the Golgi apparatus and colocalizes with *Tg*Lem3 protein, implying their occurrence as a functional complex [27]. We used conditional mutagenesis to reveal a requisite of individual *Tg*P4-ATPases (1−3) for the asexual growth of tachyzoites, which was corroborated in a subsequent study [35]. Meanwhile, in the malaria parasite, two distinct P4-ATPases and respective β-subunits were reported [36], [37]. The ATP2 flippase of *P. chabaudi*, coupling with two CDC50 homologs (*Pc*CDC50A, *Pc*CDC50B), was found to be active in the presence of PtdSer and PtdEtn [36]. The other study demonstrated a putative P4-ATPase (ATP7) in the plasma membrane and identified CDC50C as its partner protein in *P. yoelii* [37]. The complex is essential for the survival of its ookinete stage in the mosquito midgut, and PtdCho was postulated as its lipid substrate. Our earlier work discovered that the acute (tachyzoite) stage of *T. gondii* cannot import PtdCho. However, PtdSer and PtdEtn are readily internalized from the external milieu to the parasite interior [27], and any of the P4-ATPases described above may drive the process. Herein, we demonstrate that *Tg*P4-ATPase1 and *Tg*Lem1 form a functional heterocomplex to execute the flipping of PtdSer in the apical plasma membrane of tachyzoites. Our work discloses a physiological necessity of both proteins for the asexual growth of this clinically-relevant pathogen in human cells and signifies a novel function of PtdSer during the lytic cycle.

## 2 Results

### 2.1 Genetic deletion of *Tg*P4-ATPase1 and *Tg*P4-ATPase2 disrupts the lytic cycle of *T. gondii*

**Fig. 1:**
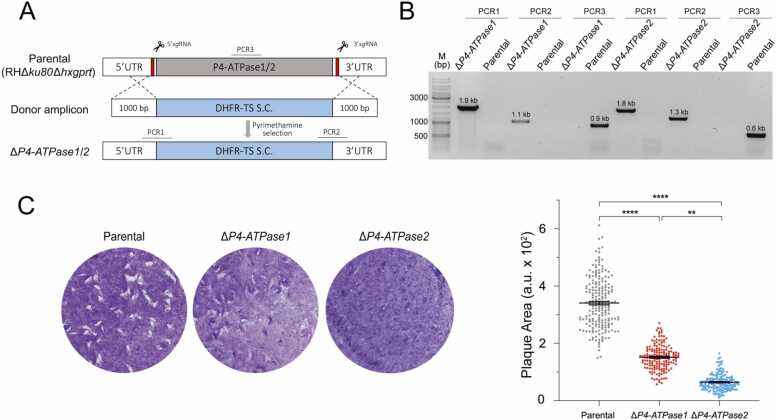
*TgP4-ATPase1 and TgP4-ATPase2 are required for the lytic cycle though not essential for the tachyzoite survival*. (A) Schematics showing the knockout strategy to generate the Δ*P4-ATPase1* and Δ*P4-ATPase2* mutants. For each strain, a vector encoding Cas9 and two *sg*RNAs targeting the 5′ and 3′ UTRs of the gene of interest (GoI) was co-transfected with a matching donor amplicon into tachyzoites of the RHΔ*ku80*Δ*hxgprt* (parental) strain. The amplicon harbored a DHFR-TS selection cassette (S.C.) flanked by 5′ and 3′ crossover sequences (1 kb). The mutants were isolated after pyrimethamine selection and following clonal dilution. (B) Genomic screening of the Δ*P4-ATPase1* and Δ*P4-ATPase2* strains using specific primers (PCR1–3, Table S1) to demonstrate the integration of the DHFR-TS cassette at the desired locus by double homologous recombination. (C) Plaque assays using the Δ*P4-ATPase1*, ∆*P4-ATPase2* and parental strains. Plaques formed by successive lytic cycles of tachyzoites appear as white areas on a host cell monolayer stained by crystal violet. For each strain, the representative images were analyzed to evaluate the area of 150–200 plaques from 3 assays (a. u., arbitrary units; means± SE; ** *p* ≤ 0.01; **** *p* ≤ 0.0001).

We have reported the conditional mutants of *Tg*P4-ATPase1–3 in tachyzoites, exhibiting a varying degree of growth defect upon protein depletion by indole-3-acetic acid (IAA, auxin) [27]. Knockdown of P4-ATPase3 imposed a lethal phenotype in plaque assays, while the lytic cycle of tachyzoites depleted in P4-ATPase1 and P4-ATPase2 was impaired by approximately 50 % and 80 %, respectively [27]. In this work, we engineered the deletion mutants of P4-ATPase1 and P4-ATPase2 (Δ*P4-ATPase1*, Δ*P4-ATPase2*). A dihydrofolate reductase – thymidylate synthase (DHFR-TS) selection cassette replaced the specified gene loci with the help of two *sg*RNAs targeting the upstream and downstream of the gene-coding region and a donor amplicon harboring 5′- and 3′-UTRs (∼1 kb) of respective P4-ATPases for double homologous recombination (Fig. 1A). The crossover events and gene deletion were verified by PCR screening of the clonal mutants using different primers (Table S1). The 5′- and 3′ recombination-specific primers yielded amplicons of the expected size (PCR1 and PCR2) in the Δ*P4-ATPase1* and Δ*P4-ATPase*2 mutants but not in the parental strain used as a negative control (Fig. 1B). Conversely, primers binding in the coding region of P4-ATPase1 and P4-ATPase2 yielded the bands (PCR3) only in the parental strain, confirming the knockout of corresponding genes.

We examined the Δ*P4-ATPase1* and Δ*P4-ATPase*2 mutants for their ability to form plaques, which signifies the successive lytic cycles of tachyzoites in a confluent host cell monolayer (Fig. 1C). Indeed, both mutants showed a significant growth impairment with respect to the parental strain. Quantification revealed ∼60 % and 85 % reduction in plaque area of the Δ*P4-ATPase1* and Δ*P4-ATPase2* strains, respectively. The effect of P4-ATPase2 deletion was more significant than the loss of P4-ATPase1. Consistent with our preceding work [27], these findings demonstrate a requirement of P4-ATPase1 and P4-ATPase2 for the lytic cycle. The fact that tachyzoites survived the deletion of individual genes and the eventual mutants could be maintained in prolonged cultures highlight unforeseen metabolic plasticity and imply a possible functional redundancy of flippases in *T. gondii*.

### 2.2 *Tg*P4-ATPase1 functions as a phosphatidylserine flippase

**Fig. 2:**
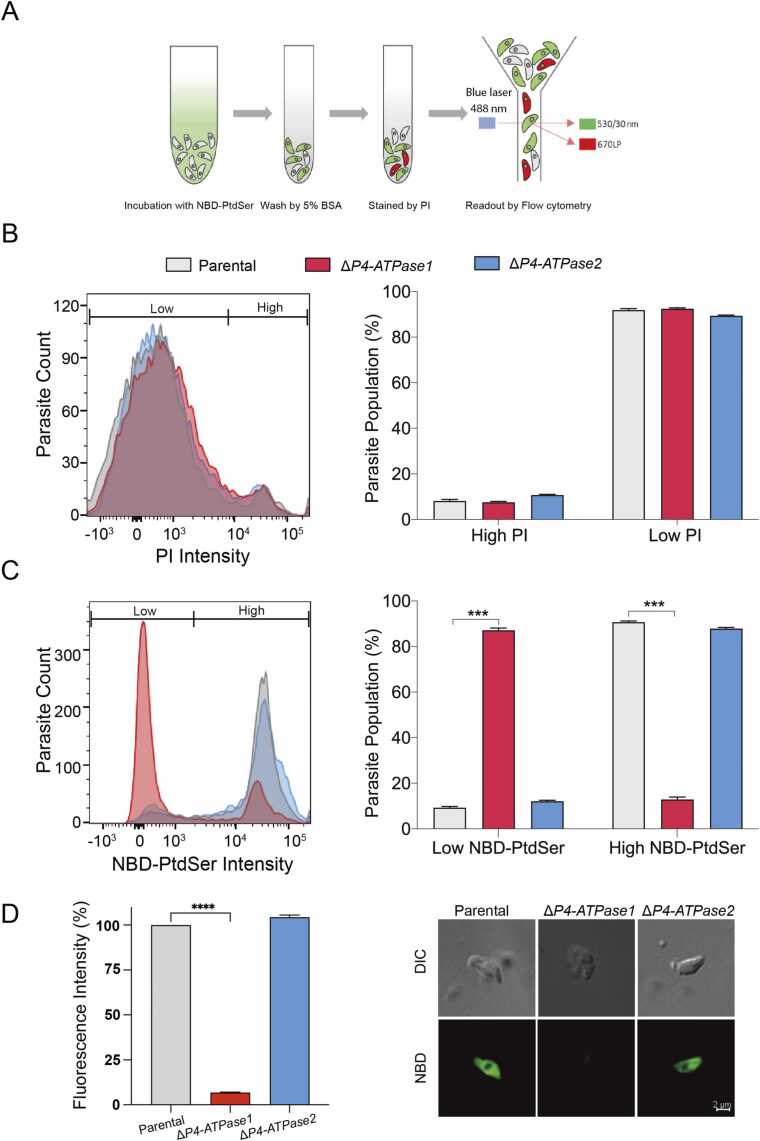
*Ablation of TgATPase1 but not TgATPase2 compromises PtdSer uptake by tachyzoites.* (A) A diagram depicting the steps of lipid uptake assay. Fresh extracellular parasites were incubated with NBD-PtdSer, followed by staining with propidium iodide (PI) and eventual flow cytometry (blue laser, 488 nm). The NBD and PI signals were detected in green (530/30 nm) and red (LP670 nm) channels, respectively. (B-C) Histograms and graphs showing the distribution of tachyzoites labeled by PI (B) and NBD-PtdSer (C). To estimate the NBD-PtdSer flipping, living tachyzoites (low PI,>90 % of the population) were analyzed in the green channel. Dead cells were excluded from the quantification. (D) NBD-PtdSer fluorescence associated with P4-ATPase1/2 mutants compared to the parental strain. Living tachyzoites were quantified irrespective of ‘low’ or ‘high’ NBD signal. Representative fluorescent images of parasites labeled with NBD-PtdSer are also shown. In *panels B-D*, curves signify one of the three independent assays, whereas graphs depict the means with standard error (*ca.* 20,000 parasites for each strain from n = 3 assays; *** *p* ≤ 0.001 **** *p* ≤ 0.0001).

P4-ATPase1 and P4-ATPase2 reside primarily in the tachyzoite’s plasma membrane [27]; thus, the knockout mutants of these proteins enabled us to examine their roles in lipid translocation activity using fluorescent probes (Fig. 2). We first examined PtdSer as a potential substrate of specified flippases because it is known to be imported by tachyzoites and then further metabolized to yield PtdEtn by PtdSer decarboxylases [27], [38], [39]. Our assays measured the internalization of NBD-PtdSer by extracellular parasites (Fig. 2A). Before flow cytometry, the lipid-labeled parasites were mixed with propidium iodide (PI). The cell population of individual samples was analyzed based on fluorescence in green (NBD-PtdSer) and red (PI) channels following excitation by a blue laser (Fig. 2A). As revealed by negligible PI staining, the parental control and the mutant strains displayed viability of about 90 % in samples (Fig. 2B), similar to freshly-harvested tachyzoites. Besides, most viable parental and Δ*P4-ATPase2* strains exhibited a strong green signal, indicating the import of NBD-PtdSer from the milieu. In noted contrast, the Δ*P4-ATPase1* strain was severely impaired in its ability to internalize the probe. Of all viable cells, only about 10 % showed a detectable NBD fluorescence, while> 90 % of the parasite population had none or weak green fluorescence (Fig. 2C). Quantifying the NBD signal in living parasites indicated a decline of> 90 % in the P4-ATPase1 mutant, whereas no change was apparent in the Δ*P4-ATPase2* strain compared to the parental control (Fig. 2D).

Next, we examined the ability of both mutants to internalize NBD-conjugated PtdEtn and PtdCho. As described elsewhere [27], the PtdEtn probe was also imported by tachyzoites (Fig S1A, S1B), while no apparent internalization of NBD-PtdCho was observed in all tested strains (Fig S2A, S2B). Neither Δ*P4-ATPase1* nor Δ*P4-ATPase2* was defective in taking up NBD-PtdEtn, precluding their role in the flipping of PtdEtn. The flow cytometry results on lipid probes were corroborated by fluorescence microscopy (Fig S1C). The Δ*P4-ATPase1* mutant was impaired in internalizing NBD-PtdSer but not the PtdEtn probe, whereas the Δ*P4-ATPase2* strain behaved akin to the parental tachyzoites (Fig. 2D, S1C). Furthermore, consistent with our preceding work [27], no signal of NBD-PtdCho was observed in any of the three strains (Fig S2C). In conclusion, these results establish that P4-ATPase1 facilitates the flipping of PtdSer on the surface of tachyzoites.

### 2.3 Conditional mutagenesis and subcellular localization of *Tg*Lem1 and *Tg*Lem3 proteins

**Fig. 3:**
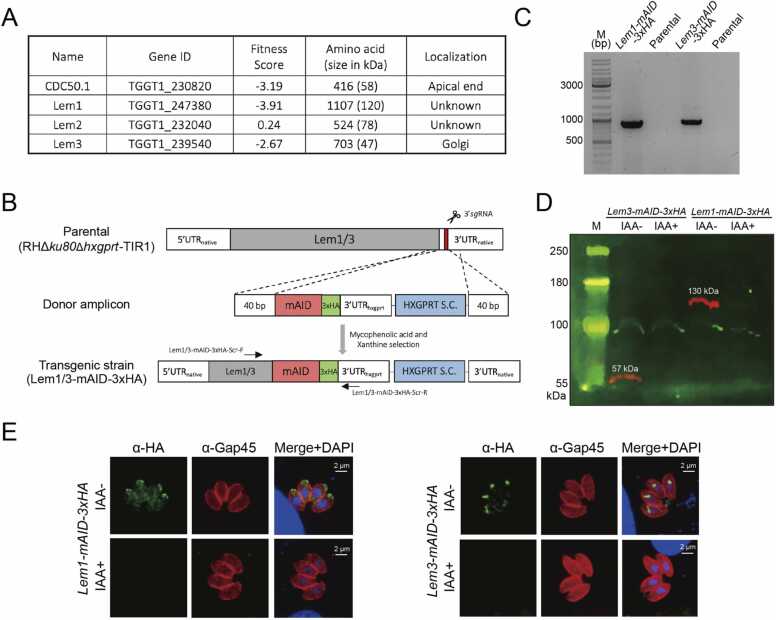
*Auxin-regulated conditional mutagenesis of Lem1 and Lem3.* (A) LEM/CDC50-like proteins (putative β-subunits of P4-ATPases) encoded by *T. gondii*. (B) Strategy for making the Lem1 and Lem3 mutants by CRISPR-Cas9-assisted 3′-genomic tagging with a mini auxin-inducible degron and 3xHA (mAID-3xHA). As indicated, the *pSAG1-Cas9-U6-TgLemX-sgRNA* constructs (X = 1 or 3) were co-transfected with respective donor amplicons into the RHΔ*ku80*Δ*hxgprt*-TIR1 strain. Tachyzoites expressing mAID-3xHA-tagged Lem1 or Lem3 proteins and HXGPRT selection cassette (S.C.) were selected. (C) Genomic PCR screening to confirm the tagging of Lem1 and Lem3 proteins with mAID-3xHA using primers indicated in *panel B* and Table S1. (D) Immunoblots of the conditional mutants before and after treatment by indole-3-acetic acid (IAA). Cell-free extract of tachyzoites (10^7^) cultured in the absence or presence of 500 μM IAA (24 h in HFFs) were resolved on 6% SDS-PAGE, followed by blotting and immunostaining with α-HA (red) and α-*Tg*Hsp90 antibodies (loading control, green). (E) Immunofluorescence assays to test the subcellular localization and auxin-dependent expression of mAID-3xHA-tagged Lem1 and Lem3 proteins. Parasites were cultured with or without 500 μM IAA for 24 h and stained with α-HA (green) and α-*Tg*Gap45 (red) antibodies. The parasite and host cell nuclei were stained with DAPI (blue).

The parasite encodes four β-subunits (CDC50.1, Lem1–3 proteins), coupling with five P4-ATPases (P4-ATPase1–5) to perform phospholipid translocation (Fig. 3A). *Tg*CDC50.1 has been characterized in the context of the cGMP signaling [29]. Herein, we focused on other orthologs, of which *Tg*Lem1 and *Tg*Lem3 show low phenotypic fitness scores (likely essential) based on a genome-wide CRISPR mutagenesis screening in the tachyzoites [40]. Our attempts to generate the knockout mutants of these proteins were unsuccessful, indicating their physiological necessity for the parasite survival. To consolidate this notion, we performed 3′- genomic tagging of Lem1 and Lem3 with a mini auxin-inducible degron and 3xHA (mAID-3xHA) [41] (Fig. 3B). Tachyzoites expressing mAID-3xHA-tagged Lem1 or Lem3 were selected using mycophenolic acid and xanthine, and then clonal parasites were screened by genomic PCR (Fig. 3B,3C). As depicted for the representative clones, we observed an expected amplicon (∼950 bp) in the *Lem1-mAID-3xHA* and *Lem3-mAID-3xHA* mutants but not in the parental (RHΔ*ku80*Δ*hxgprt*-TIR1) strain (Fig. 3C). Besides, both Lem1 and Lem3 proteins could be depleted by indole-3-acetic acid (IAA, auxin) in respective mutants, as shown by the disappearance of ∼130-kDa and ∼57-kDa bands in immunoblots, respectively (Fig. 3D). Immunofluorescence assay also confirmed the IAA-induced knockdown of Lem1 and Lem3 (Fig. 3E). Moreover, the apical presence of Lem1-mAID-3xHA, as verified by its colocalization with inner membrane complex marker protein *Tg*ISP1 [42] (Fig S3A), suggested its coupling with P4-ATPase1 (*see below for additional results*). Whereas, Lem3 localized in the Golgi network, as reported earlier [27], indicating its possible partnership with P4-ATPase3. Furthermore, Lem1 was not detectable in the budding daughter cells (Fig S3B), as judged by staining with *Tg*IMC3, a marker of inner membrane complex during endodyogeny [43].

### 2.4 Depletion of *Tg*Lem1 compromises the parasite growth and internalization of PtdSer

**Fig. 4:**
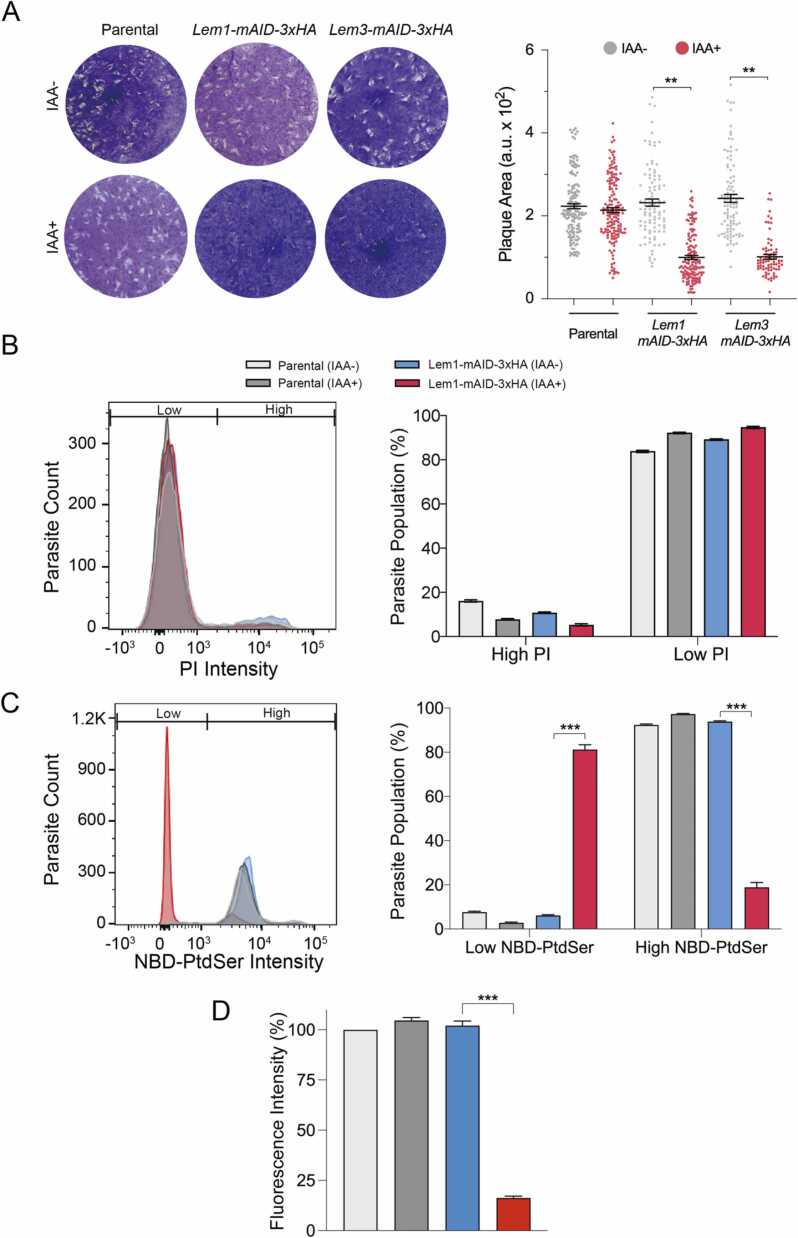
*Conditional depletion of Lem1 and Lem3 impairs the lytic cycle, and the former protein is involved in PtdSer flipping at the parasite surface*. (A) Plaques formed by mAID-3xHA-tagged Lem1 and Lem3 mutants and parental (RHΔ*ku80*Δ*hxgprt*-TIR1) strain. Images show crystal violet-stained host cell monolayer (blue) with plaques formed by sequential lytic cycles (white area). The scatter graph displays the area of 150–200 plaques in arbitrary units (a. u.) for each strain (n = 3, means± SE, ** *p* ≤ 0.01). (B-C) Flow cytometry of NBD-PtdSer import by *Lem1-mAID-3xHA* and parental strains. The lipid flipping assay was set up as illustrated in Fig. 2A. Histograms and graphs show the distribution of PI- and NBD-stained tachyzoites, precultured without or with indole-3-acetic acid (500 μM IAA, 24 h in HFFs). (D) NBD-PtdSer fluorescence associated with Lem1 mutant compared to the parental strain under -/+IAA conditions. Living tachyzoites irrespective of ‘low’ or ‘high’ NBD signal were quantified. In *panels B-D*, curves represent one of the three assays, while graphs show the mean values with standard error (*ca.* 20,000 parasites/strain, n = 3 assays, *** *p* ≤ 0.001). To calculate the NBD-PtdSer internalization, only living tachyzoites (low PI,>90 % cells) were analyzed.

We next tested the relative growth of the mAID-3xHA-tagged Lem1 and Lem3 mutants by plaque assays in the absence or presence of auxin (Fig. 4A). Both mutants displayed significantly reduced plaques formed in the HFF monolayers compared to the parental strain. Indeed, the average plaque size was much smaller (∼40 % of the parental control) after the depletion of Lem1 and Lem3 by IAA. The phenotype of the *Lem1-mAID-3xHA* mutant and apical location (Fig. 3E) implied its interaction with P4-ATPase1 and, thereby, a function in PtdSer flipping, which prompted us to test the conditional strain with NBD-PtdSer probe (Fig. 4B-4D), as described above (Fig. 2A). The viability of samples was about 85 % (Fig. 4B). We observed that> 80 % of the parental and *Lem1-mAID-3xHA* tachyzoites could import NBD-PtdSer from their environment when cultured in the absence of IAA. While exposure to IAA did not impact the control strain, it caused a significant decline in NBD-positive cells of the Lem1 mutant (Fig. 4C). A similar observation was made when NBD fluorescence intensity associated with living tachyzoites was quantified for all samples (Fig. 4D). Overall, these results show the importance of Lem1 for the lytic cycle and advocate its role in PtdSer flipping, possibly by coupling with P4-ATPase1.

### 2.5 The Δ*P4-ATPase1* and *Lem1-mAID-3xHA* mutants phenocopy the lytic cycle defects

**Fig. 5:**
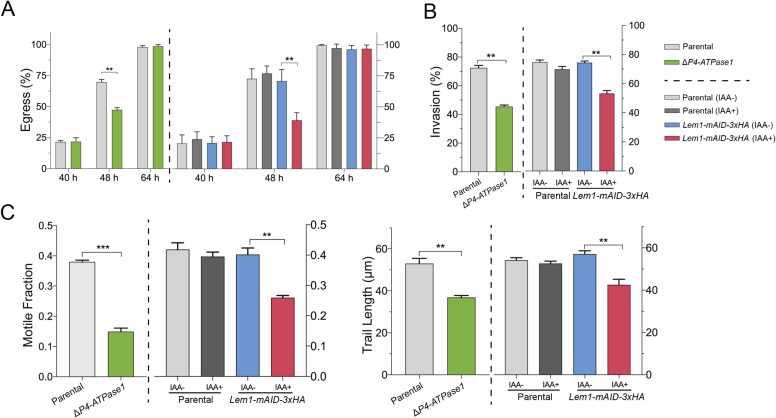
*The* Δ*P4-ATPase1 and Lem1-mAID-3xHA strains phenocopy each other.* (A-C) Detailed phenotyping of the P4-ATPase1 and Lem1 mutants along with related parental strains. Graphs show the parasite egress at 40 h, 48 h and 64 h post-infection (A), host-cell invasion (B) and gliding motility (C) using standard immunostaining methods. About 1000 parasites of each strain from four assays were examined to estimate the invasion efficiency. The natural egress of tachyzoites was measured by scoring 500–600 vacuoles/strain (n = 3 assays). Where indicated, IAA was added 24 h post-infection. For gliding motility, immunostained tachyzoites from three experiments were evaluated for the motile fraction (500 parasites/strain) and trail lengths (100–120 trails/strain). Tachyzoites were precultured for 24 h without or with IAA before the invasion and motility assays were set up. Graphs in *panels A-C* show data from 3 assays (means± SE, ** *p* ≤ 0.01, *** *p* ≤ 0.001).

Impaired growth of the Δ*P4-ATPase1* and *Lem1-mAID-3xHA* strains encouraged us to examine their physiological importance for individual steps of the lytic cycle, including replication, invasion, egress, and gliding motility (Fig. 5, S4). The parental strains corresponding to each mutant served as the controls in comparative phenotyping. To begin with, no difference in the replication of tachyzoites, as gauged by the size of parasitophorous vacuoles (number of progeny/vacuole) at 24 h and 40 h post-infection, was apparent in the mutants (Fig S4). In contrast, the loss of P4-ATPAse1 and depletion of Lem1 negatively impacted the egress and invasion at similar levels in both mutants (Fig. 5A,5B). Notably, the egress defect was recorded only after 48 h of infection in both mutants (Fig. 5A). All strains eventually exited with comparable efficiency in late-stage cultures (64 h infection). The Δ*P4-ATPase1* and *Lem1-mAID-3xHA* strains were also impaired in invading host cells (Fig. 5B). Moreover, the egress and invasion defects were recapitulated in the gliding motility assays (Fig. 5C). The two mutants displayed significantly lower motile fractions and trail lengths than the respective control (parental) strains. Collectively, these findings underline the specific need for P4-ATPase1 and Lem1 for the motility-dependent egress and invasion events, although not for the intracellular replication, during the lytic cycle of *T. gondii*.

### 2.6 Proximity-dependent biotin labeling reveals P4-ATPase1/Lem1 interaction in tachyzoites

**Fig. 6:**
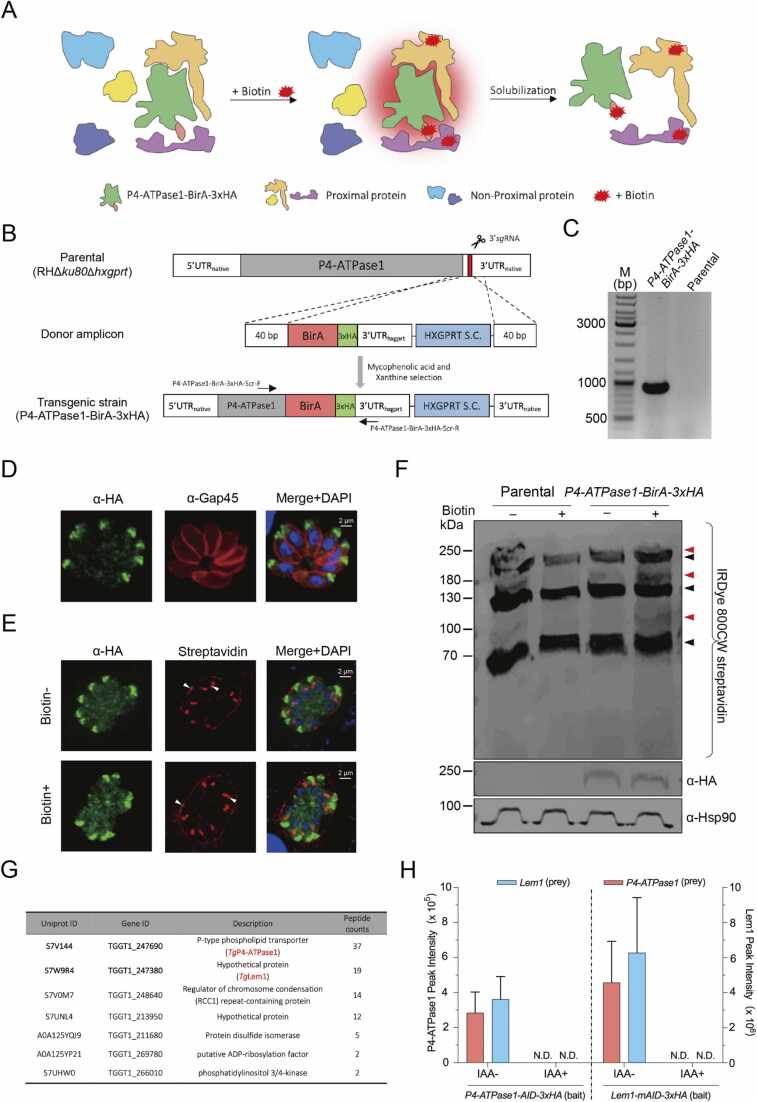
*P4-ATPase1 and Lem1interact with each other to form a functional complex*. (A) A diagram illustrating the proximal biotinylation of interaction partners by a biotin ligase A (BirA) fused to P4-ATPase1. Biotinylated proteins were solubilized and captured by streptavidin beads. (B) Schematics of CRISPR-Cas9-aided C-terminal tagging of P4-ATPase1 with the BirA-3xHA epitope. The *pSAG1-Cas9-U6-TgP4-ATPase1-sgRNA* construct was transfected with a donor amplicon (BirA-3xHA-3′UTR_Gra1_-HXGPRT . flanked by 5′ and 3′ crossover sequences) into the parental strain (RHΔ*ku80*Δ*hxgprt*). The *P4-ATPase1-BirA-3xHA* strain was selected for the HXGPRT selection cassette (S.C.). (C) Genomic PCR confirming the BirA-3xHA tagging of P4-ATPase1 using primers shown in *panel B* and Table S1 (D) Tachyzoites expressing P4-ATPase1-BirA-3xHA after immunostaining of the 3xHA tag and *Tg*Gap45 (inner membrane complex marker). (E) Images of tachyzoites (*P4-ATPase1-BirA-3xHA*) precultured in the absence or presence of biotin (150 μM, 20 h), followed by staining with α-HA and streptavidin. Arrowheads show the biotinylated proteins. Specifically, streptavidin labeling of acetyl-CoA carboxylase in the parasite apicoplast and pyruvate carboxylase in the host mitochondria are evident (note the staining of the parasitophorous vacuole membrane due to recruitment of the host mitochondria). (F) Immunoblot of the parental (RHΔ*ku80*Δ*hxgprt*) and *P4-ATPase1-BirA-3xHA* strains under (-) and (+) biotin conditions. IRDye 800CW-streptavidin was used to detect biotinylated proteins, whereas α-HA and α-*Tg*Hsp90 antibodies were deployed to visualize expression of P4-ATPase1-BirA-3xHA and *Tg*Hsp90 (loading control). Protein bands shared by the parental and mutant strains indicate natural biotinylated proteins (black triangles), whereas those detected mainly in the biotin-treated P4-ATPase1-BirA-3xHA strain show potential interactors (red triangles). (G) Interactors of P4-ATPase1, identified after mass spectrometry of the biotin-labeled *P4-ATPase1-BirA-3xHA* strain. Seven proteins, including P4-ATPase1 and Lem1, were significantly enriched in the *P4-ATPase1-BirA-3xHA* strain compared to the parental control. For additional details, refer to Table S2. (H) Mass spectrometric detection of Lem1 and P4-ATPase1 proteins in *P4-ATPase1-AID-3xHA* and *Lem1-mAID-3xHA* strains, after reciprocal immunoprecipitation using α-HA agarose beads. IAA treatment was performed for 24 h in parasite cultures before sample collection. Note the absence of the bait and, thereby, the partner protein in IAA-treated samples (N.D., not detectable).

To examine the predicted cooperation of P4-ATPase1 and Lem1 proteins, we deployed a proximity-dependent biotinylation method utilizing a biotin ligase (BirA) of *E. coli* [44] (Fig. 6A). We conjugated P4-ATPase1 with the BirA-3xHA epitope *via* CRISPR/Cas9-aided 3′- genomic tagging in tachyzoites, as described previously [45] (Fig. 6B). In this regard, a donor amplicon carrying BirA-3xHA and the HXGPRT cassette flanked by 5′ and 3′ homology arms of P4-ATPase1 were co-transfected along with a vector encoding Cas9 and 3′UTR-specific *sg*RNA into RHΔ*ku80*Δ*hxgprt* strain. Parasites were selected with mycophenolic acid and xanthine (Fig. 6B), and positive clones harboring P4-ATPase1-BirA-3xHA fusion were confirmed by genomic screening using recombination-specific primers (Fig. 6C, Table S1). The apical presence of P4-ATPase1-BirA-3xHA shown by immunostaining excluded any transgenic artifact due to genomic tagging (Fig. 6D). Supplementation of the parasite culture with biotin allowed the BirA domain to biotinylate proteins proximal to P4-ATPase1 and thereby marked potential partner proteins (Fig. 6A). The parental strain in the absence or presence of biotin was included as a control.

Immunofluorescence staining of the *P4-ATPase1-BirA-3xHA* strain by α-HA antibody showed a robust apical signal irrespective of biotin (Fig. 6E). On the other hand, streptavidin stained the apicoplast, mitochondrion and parasitophorous vacuole membrane, corresponding to the biotin-dependent acetyl-CoA carboxylase [46], pyruvate carboxylase [47] and vacuole-associated host mitochondrial proteins, respectively. The *P4-ATPase1-BirA-3xHA* and parental strains were subjected to pulldown of the biotinylated proteins by streptavidin-coated magnetic beads. As expected, the α-HA-immunoblot confirmed the expression of P4-ATPase1-BirA-3xHA (∼243-kDa) (Fig. 6F). Two other bands matching with the acetyl-CoA carboxylase and pyruvate carboxylase were observed in both strains regardless of the cofactor (Fig. 6F, black triangles). Besides, immunoblot pointed to additional bands in biotin-treated *P4-ATPase1-BirA-3xHA* samples compared to untreated and parental controls (Fig. 6F, red triangles). The mass spectrometry analysis detected> 170 proteins, including naturally biotinylated proteins across all samples, of which only seven proteins, including P4-ATPase1 and Lem1, were identified exclusively in the *P4-ATPase1-BirA-3xHA* strain (Fig. 6G, Table S2). In conjunction with the above data, our proximity-dependent labeling assay strongly suggests the occurrence of a functional complex between the two proteins in tachyzoites of *T. gondii*.

### 2.7 Reciprocal immunoprecipitation endorses the physical association of P4-ATPase1 and Lem1

Encouraged by the aforementioned datasets, we set up reciprocal immunoprecipitation assays using the existing strains (*P4-ATPase1-AID-3xHA* [27] and *Lem1-mAID-3xHA* (Fig. 3)) in the presence or absence of IAA. The protein samples were analyzed by liquid chromatography-mass spectrometry (LC-MS). As shown (Fig. 6H), we detected Lem1 in α-HA-precipitated proteins of the *P4-ATPase1-AID-3xHA* strain cultured in the absence of IAA (*on-state*, P4-ATPase1-replete) but not in the presence (*off-state*, P4-ATPase-depleted). Similarly, we found P4-ATPase1 in samples after α-HA-pulldown of the *Lem1-mAID-3xHA* strain incubated without IAA (Lem1-replete) but not when exposed to the compound (Lem1-depleted). As expected, the bait proteins were detected in both mutants (Fig. 6H). These experiments provide strong evidence of physical interaction between the two proteins for PtdSer translocation during the lytic cycle of *T. gondii*.

## 3 Discussion

**Fig. 7:**
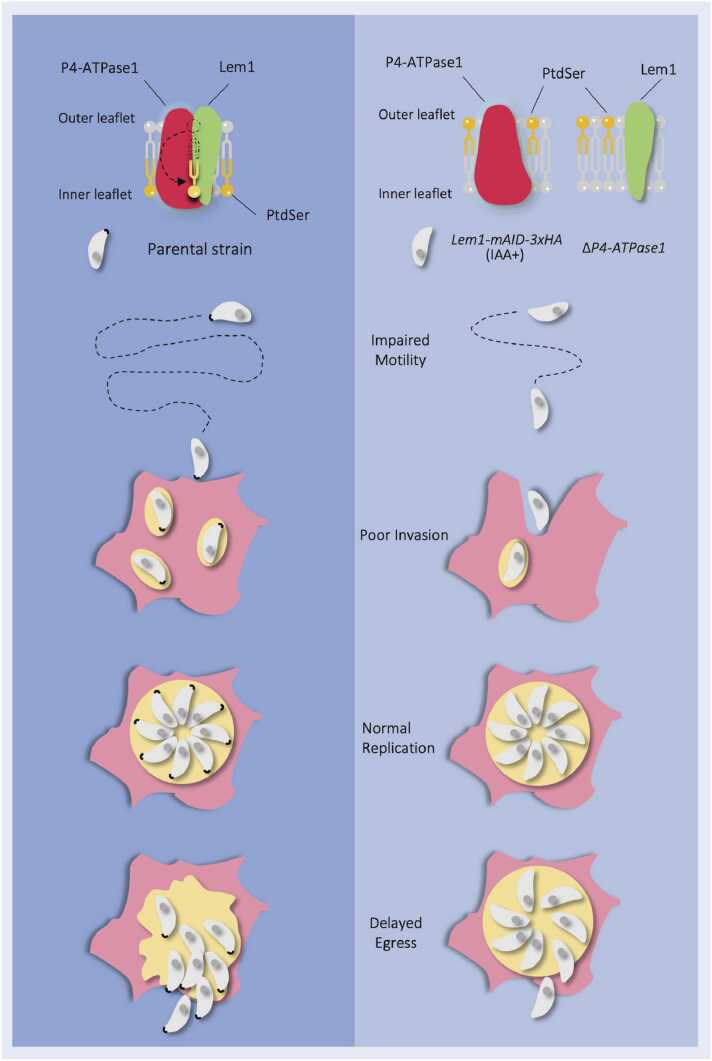
*PtdSer flipping by P4-ATPase1-Lem1 complex and its physiological importance during the lytic cycle of T. gondii*. As shown, in healthy parasites, P4-ATPase1 and Lem1 form a heterocomplex involved in PtdSer flipping at the apical plasma membrane, which facilitates the motility-dependent egress and invasion events (*left*). Disruption of either protein impairs the lipid translocation and the lytic cycle (*right*), while the parasite replication is unaffected in any of the two mutants.

Our prior work has demonstrated the translocation of PtdSer and PtdEtn into tachyzoites, identified five P4-ATPases and three LEM/CDC50-family proteins and disclosed the physiological importance of P4-ATPase1–3 for the lytic cycle of *T. gondii* [27]. Here, we show the P4-ATPase1-Lem1 complex, located at the invasive (apical) pole of tachyzoites, as a significant player in PtdSer flipping and motility-dependent egress and invasion (Fig. 7). Besides, this study reveals that P4-ATPase1 and P4-ATPase2 are required for a normal lytic cycle but dispensable for the survival of tachyzoites. Both proteins are considered to be essential according to a genome-wide phenotypic screening [40] and based on conditional mutagenesis in a recent study [35], overlapping with our previous report [27] and this work. We demonstrate that tachyzoites can cope with the individual deletion of P4-ATPase1 and P4-ATPase2. The corresponding mutants continue to grow in prolonged cultures, underlining the unexpected and previously-unknown plasticity of phospholipid flipping in *T. gondii*. Any functional redundancy and synthetic lethality of P4-ATPases warrant combinative mutagenesis in tachyzoites.

P4-ATPase1 can internalize PtdSer but not PtdEtn or PtdCho; whether it can facilitate the import of other phospholipids remains to be investigated. In this context, we have described the occurrence of phosphatidylthreonine (PtdThr) – a natural homolog of otherwise-universal PtdSer – in *T. gondii* and *Eimeria falciformis* [24], [26], [48], [49]. Notably, the deletion of PtdThr synthase phenocopies the P4-ATPase1 and Lem1 mutants and displays a perturbed calcium homeostasis [24], a well-known trigger for motility-dependent egress and invasion events. It is thus plausible that the P4-ATPase1-Lem1 complex controls the calcium-dependent events by PtdThr flipping in *T. gondii*. Testing this premise requires the synthesis of PtdThr and its fluorescent probes (not commercially available). Similarly, additional lipid probes, *e.g.*, ceramide, glucosyl-/galactosyl-ceramide, and sphingolipids, remain to be tested as potential substrates of P4-ATPase1 and P4-ATPase2 proteins. Interestingly, PtdEtn is readily internalized by the parental parasites [27]; however, neither Δ*P4-ATPase1* nor Δ*P4-ATPase2* mutant exhibited an impairment in its flipping, suggesting its uptake *via* other P4-ATPases or endocytosis-like pathway that is also present in apicomplexan parasites [50]. The leaflet translocation of PtdSer and PtdEtn by Drs2p–Cdc50p in *Saccharomyces cerevisiae* is dependent on the conserved **N**, **P** and **A** domains, as well as on a regulatory (**R**) sequence containing the GYAFS motif in Drs2p, which is critical for the inhibition/activation of the flippase complex by phosphatidylinositol-4-phosphate [17], [18]. This motif is also conserved in P4-ATPase1 [27], implying a similar *modus operandi* for PtdSer flipping in tachyzoites of *T. gondii*.

Most orthologs of apicomplexan P4-ATPases in mammalian cells and yeast are known to interact with a non-catalytic β-subunit (LEM/CDC50) for their flippase function. Similarly, we show that Lem1 and Lem3, located in the apical region and the Golgi network, respectively, are crucial for the parasite growth; and the knockdown of the former protein phenocopies the P4-ATPase1 mutant (Fig. 7). Our work shows the interaction of P4-ATPase1 with Lem1 and suggests coupling of P4-ATPase3 with Lem3, likely in the form of a functional heterocomplex. As LEM/CDC50 proteins can interact with more than one α-subunits [51], hence it is plausible that *Tg*Lem1 may also associate with other P4-ATPases and thereby contribute to the translocation of other phospholipids in tachyzoites. Besides Lem1, we found a few other proteins interacting with P4-ATPase1, including a phosphatidylinositol 3/4-kinase, which remained to be examined in the future.

The endocytic-like structures have been described and known to internalize lipids in intracellular tachyzoites [52]. Likewise, lipid probes can also be endocytosed by extracellular parasites, although trafficking factors remain poorly understood [53]. Thus, we cannot rule out the role of endocytosis in phospholipid internalization; however, the observed defect in translocation of PtdSer but not of PtdEtn in the Δ*P4-ATPase1* mutant and selectivity of lipid uptake in tachyzoites demonstrate the functional importance of P4-ATPase1-Lem1 heterocomplex in *T. gondii*. Accordingly, reduction in the motility, invasion and egress in both Δ*P4-ATPase1* and *Lem1-mAID-3xHA* mutants point to a role of the P4-ATPase1-Lem1 complex and PtdSer in exocytosis of secretory organelles [35]. Whether and how PtdSer, PtdThr and/or any other lipid regulate the observed phenotype remains to be examined. In conclusion, the shared apical location, flipping activity, phenotypic traits and physical association strongly advocate a functional coupling of P4-ATpase1 and Lem1 as α and β subunits in tachyzoites. Besides, a physiological requirement of both proteins underscores the therapeutic potential of PtdSer flipping during acute toxoplasmosis.

## 4 Experimental procedures

### 4.1 Biological resources and reagents

Human foreskin fibroblast (HFF) cells to maintain tachyzoites of *T. gondii* were provided by Carsten Lüder (George-August University, Göttingen). The RHΔ*ku80*Δ*hxgprt* strain lacking hypoxanthine-xanthine-guanine phosphoribosyltransferase (Δ*hxgprt*) as well as the nonhomologous end-joining DNA repair (Δ*ku80*) was offered by Vern Carruthers (University of Michigan, USA) [54], [55], [56]. The RHΔ*ku80*Δ*hxgprt*-TIR1 strain and the plasmids (*pLinker-mAID-3xHA-HXGPRT*, *pLinker-BirA-3xHA-HXGPRT*) were gifted by David Sibley (Washington University, USA) [41]. The *pUC19* vector for making the gene-specific donor amplicons for 5′ and 3′ recombination was provided by Bang Shen (Huazhong Agricultural University, Wuhan China) [57]. The primary antibodies binding *Tg*Gap45 and *Tg*Hsp90 proteins were provided by Dominique Soldati-Favre (University of Geneva, Switzerland) and Sergio Angel (IIB-INTECH, Buenos Aires, Argentina), respectively. Antibodies against *Tg*ISP1 and *Tg*IMC3 were offered by Peter Bradley (University of California, Los Angeles, USA) and Marc-Jan Gubbels (Boston College, MA, USA). Other primary antibodies recognizing the HA epitope and *Tg*Sag1 were procured from Takara-Bio (Japan) and Sigma-Aldrich (Germany). The secondary antibodies (Alexa488, Alexa594; IRDye 680RD, 800CW) and oligonucleotides (Table S1) were obtained from ThermoFisher Scientific (Germany). The cell culture media and additives were purchased from PAN Biotech (Germany), and other standard chemicals were supplied by Sigma-Aldrich and Carl Roth (Germany). The reagent kits for isolation, cloning and purification of nucleic acids were acquired from Analytik Jena and Life Technologies (Germany). Lipids tagged with C6-nitrobenzoxadiazole (NBD) were acquired from Avanti Polar Lipids (USA).

#### 4.1.1 Parasite and host cell culture

Host cells were harvested by trypsinization and grown to confluence in flasks, dishes, and plates. Uninfected and parasitized HFFs were cultured in a humidified incubator (5 % CO_2_, 37 °C) using Dulbecco’s Modified Eagle’s Medium supplemented with glucose (4.5 g/L), fetal bovine serum (10 %), glutamine (2 mM), sodium pyruvate (1 mM), penicillin (100 U/mL), streptomycin (100 μg/mL), and Eagle’s minimum nonessential amino acids. Unless stated otherwise, HFFs were infected with a defined multiplicity of infection (MoI, 2–3 parasites/host cell). For most assays, parasites were harvested by squirting the late-stage parasitophorous vacuoles (40–44 h infection) through a 27 G syringe (3 times) and washed with phosphate-buffered saline. Extracellular tachyzoites were used immediately after isolation for all downstream experiments.

#### 4.1.2 Generation of P4-ATPase knockout mutants

The P4-ATPase genes were replaced by a dihydrofolate reductase-thymidylate synthase (DHFR-TS) selection cassette. The process of double homologous recombination at the targeted locus was facilitated by a dual-CRISPR-Cas9 plasmid expressing Cas9 and two *sg*RNAs binding to the 5′ UTR and 3′ UTR regions of respective P4-ATPase genes. The vector was generated by Q5 site-directed mutagenesis kit (New England Biolabs). The 5′ UTR (1 kb before the start codon) and 3′ UTR (1 kb after the stop codon) of P4-ATPase1 and P4-ATPase2 were amplified from the genomic DNA and cloned into the *pUC19* vector flanking the DHFR-TS selection cassette using a Gibson assembly kit (Vazyme Biotech). The locus-specific donor amplicons were generated from the *pUC19* constructs and co-transfected along with the matching CRISPR-Cas9 vectors encoding 5′*sg*RNA and 3′*sg*RNA for P4-ATPases into the RHΔ*ku80*Δ*hxgprt* strain. Tachyzoites expressing DHFR-TS were selected by 1 μM pyrimethamine [58] and cloned by limiting dilution in 96-well plates. The knockout mutants were screened by crossover-specific genomic PCR.

#### 4.1.3 Making of auxin-inducible Lem1 and Lem3 mutants

To engineer the conditional mutant of the β-subunits, we implemented an auxin-induced degradation method [41]. The CRISPR-Cas9 constructs were desidgned with 3′-UTR-specific *sg*RNA of Lem1 or Lem3 (∼100 bp after the stop codon). The corresponding donor amplicons, comprising the mAID-3xHA epitope and HXGPRT expression cassette flanked by 5′ and 3′ homology arms (40 bp), were amplified using the Phanta Max Super-Fidelity DNA polymerase (Vazyme Biotech, China) and *pLinker-mAID-3xHA-HXGPRT* plasmid template. The matching donor amplicon and *sg*RNA-carrying vectors were co-transfected into the RHΔ*ku80*Δ*hxgprt*-TIR1 tachyzoites, followed by drug selection (mycophenolic acid, 25 μg/mL; xanthine, 50 μg/mL), as reported elsewhere [59]. The epitope tagging of Lem1 and Lem3 was verified by crossover-specific genomic PCR of the clonal strains, sequencing and immunostaining assays.

#### 4.1.4 Immunoblot analysis

Tachyzoites (2 × 10^7^) were harvested, washed with ice-cold PBS, and pelleted (3000 *g*, 5 min, 4 °C). Cells were lysed on ice for 30 min in 50 μL lysis buffer (10 mM K_2_HPO_4_, 150 mM NaCl, 5 mM EDTA, 5 mM EGTA, pH 7.4; 0.2 % sodium deoxycholate, 1 % Triton X-100) containing protease inhibitors (Sigma). Samples were centrifuged (20,000 *g*, 15 min, 4 °C) to collect the supernatant, mixed with the Laemmli buffer and resolved by SDS-PAGE (6 %). Proteins were transferred onto a nitrocellulose membrane (85 mA/cm^2^, 2 h) and stained by mouse α-HA (1:10,000) and rabbit α-*Tg*Hsp90 (1:10,000) antibodies diluted in 5 % skimmed milk with 0.2 % Tween 20/TBS-T. The membrane blot was washed 3x with 0.2 % Tween 20/TBS-T (5 min) and incubated with IRDye-conjugated antibodies (680RD and 800CW, 1:3000) for 1 h. The protein bands were visualized by an Odyssey Fc imaging system (LI-COR Biosciences).

#### 4.1.5 Indirect immunofluorescence assay

HFFs grown on glass coverslips (thickness, 1.5 mm; 12 mm diameter) were infected with tachyzoites (MoI, 2; 24–44 h). Cultures were washed with 500 μL PBS, fixed in 4 % paraformaldehyde/PBS (15 min), and neutralized by 0.1 M glycine/PBS solution (500 μL, 5 min). Samples were permeabilized in 1 mL of 0.2 % Triton X-100/PBS (20 min), followed by blocking in 500 μL PBS containing 2 % BSA (w/v) and 0.2 % Triton X-100 (20 min). Cells were then treated with primary antibodies in a blocking solution (mouse or rabbit α-HA, 1:3000; rabbit α-*Tg*Gap45, 1:10000; rabbit α-*Tg*ISP1, 1:2000; rabbit α-*Tg*IMC3, 1:2000; 1 h, room temperature). Coverslips were washed 3x with 1 mL of 0.2 % Triton X-100 in PBS (5 min) and stained by fluorophore-conjugated secondary antibodies (Alexa488, 1:3000 and Alexa594, 1:3000; 45 min in the dark). Samples were washed 3x with PBS and mounted in Fluoromount G/DAPI for fluorescent imaging (Zeiss, Germany).

#### 4.1.6 Parasite phenotyping assays

Plaque assays were performed to examine the overall fitness of the mutants in confluent HFF cells. Host cells were infected (200 tachyzoites/well) and incubated for seven days under standard culture conditions without any perturbation. Samples were fixed with ice-cold methanol (−80 °C, 10 min) and stained with crystal violet dye for 15 min, followed by PBS washing. Plaques were imaged, and their size/area was measured using Adobe Photoshop (2020 suite). To quantify the replication rate, HFFs grown on coverslips were infected (10^5^ parasites, 24 h and 40 h), followed by fixation, neutralization, permeabilization, blocking and staining with the α-*Tg*Gap45 and Alexa594 antibodies (see above). The cell division was scored by counting parasites developing within their parasitophorous vacuoles.

For the motility assay, tachyzoites (4 x 10^5^) were suspended in calcium- and magnesium-free Hank’s balanced salt solution (HBSS) and centrifuged (300 *g*, 5 min, 37 °C) to settle them on the BSA-coated (0.01 % in PBS) coverslips in a 24-well plate. Samples were incubated in a humidified incubator (15 min, 37 °C), followed by fixation in 4 % paraformaldehyde/PBS (15 min) and neutralization with 0.1 M glycine/PBS (5 min). Samples were blocked in 3 % BSA/PBS (30 min) and immunostained by mouse α-*Tg*Sag1 (1:200) and Alexa488 antibodies prepared in the blocking buffer (1 h). The fraction of moving parasites was counted by a microscope, whereas the average trail lengths were quantified using the ImageJ program.

The invasion and egress assays were executed, as described previously [31]. Briefly, host-cell monolayers cultured on glass coverslips were infected (MoI: 10, 1 h, invasion assay) (MoI: 1 for 40, 48, 64 h, egress assay). Cells were fixed with 4 % paraformaldehyde/PBS (15 min), neutralized by 0.1 M glycine/PBS (5 min), and then blocked in 3 % BSA/PBS (30 min). Non-invaded and egressing tachyzoites (extracellular stage) were stained by α-*Tg*Sag1 antibody (mouse, 1:200, 1 h) before membrane permeabilization. Samples were then permeabilized by 0.2 % Triton-X 100 in PBS (20 min) and stained by α-*Tg*Gap45 antibody (1:8000, 1 h) to visualize intracellular tachyzoites. After 3x PBS washing, coverslips were incubated with secondary antibodies (Alexa488, Alexa594, 1:3000, 1 h). The fraction of invaded parasites was determined by counting tachyzoites labeled with α-*Tg*Gap45/Alexa594 (red). On the other hand, the fraction of lysed vacuoles (egressing parasites) was scored by enumerating dual-colored tachyzoites.

#### 4.1.7 Immunoprecipitation of P4-ATPase1 and Lem1

The immunoprecipitation was executed, as described previously [60]. The native *Tg*P4-ATPase1-smHA and *Tg*Lem1-mAID-3xHA proteins were subjected to pulldown by using the monoclonal α-HA agarose beads (HA-7, Sigma Aldrich, Germany). Cell-free extract was prepared as reported in *Immunoblot Analysis*, mixed with 25–50 μL of agarose beads, and the volume was adjusted to 1 mL by adding lysis buffer supplemented with protease inhibitor cocktail (trypsin, 20 mg/mL; aprotinin, 10 mg/mL; benzamidine, 500 mg/mL; PMSF, 0.5 mM; Na_3_VO_4_, 0.1 mM; NaF, 50 mM). Subsequent to incubation with constant shaking (6 h, 4 °C), beads were pelleted (200 *g*, 30 *s*), washed once with ice-cold lysis buffer with protease inhibitor cocktail and then twice with PBS. The immunoprecipitated proteins were analyzed by mass spectrometry in data-independent acquisition (DIA) mode.

#### 4.1.8 NBD-lipid internalization assay

The experiment was performed essentially as described previously [27]. The parasitized HFFs (MoI, 2; 40–44 h infection) were scraped in the culture medium and squirted through a 27 G syringe (3x) to release tachyzoites. Parasites were washed 3x with PBS (1400 *g*, 10 min, 4 °C) and then suspended in cold labeling medium (950 μL/sample, 20 mM HEPES, 140 mM KCl, 10 mM NaCl, 2.5 mM MgCl_2_, 0.1 μM CaCl_2_, 1 mM sodium pyruvate, 1 mM ATP, 5 mM glucose, 1x MEM vitamins, and 1x serine-free non-essential amino acids; pH 7.4). The NBD-lipid probes (1 nmol/sample) were dried in conical-bottom glass tubes, resuspended in ethanol (5 μL/sample) and then mixed with the labeling medium (50 μL/sample). The assay (10^7^ parasites and NBD-lipid in 1 mL) was performed in a shaking water bath (37 °C, 30 min). Samples were quenched on ice, washed 2x with 5 % BSA (1400 *g*, 10 min, 4 °C) and then 2x with the labeling medium to eliminate the probe incorporated into the exoplasmic leaflet of the plasma membrane. The parasite pellets were eventually suspended in 500 μL PBS for flow cytometry (FACScan, Becton Dickinson) to analyze the lipid translocation to the cytoplasmic leaflet. Each NBD-labeled sample (500 μL) was mixed with 1 μL propidium iodide (1 μg/mL), and 10,000–20,000 tachyzoites were examined using green (530/30 nm) and red (LP670 nm) channels. Living cells were selected by propidium iodide exclusion (low PI) and analyzed for NBD-lipid labeling. Data were analyzed using the CellQuest and Cyflogic software (CyFlo Ltd).

#### 4.1.9 BirA-mediated proximity labeling

As reported previously, we applied the proximity-dependent biotin identification (BioID) method [45] to search for P4-ATPase1-interaction partners. A plasmid expressing Cas9 and *sg*RNA to target the P4-ATPase1–3′ UTR was constructed (see primers in Table S1). The donor amplicon – BirA-3xHA epitope and HXGPRT expression cassette flanked by 5′ and 3′ of homology arms of P4-ATPase1 (40 bp) – was amplified using the Phanta Max Super-Fidelity DNA polymerase (Vazyme Biotech, China) and the *pLinker-BirA-3xHA-HXGPRT* template (Table S1). The CRISPR plasmid and donor amplicon were co-transfected into the RHΔ*ku80*Δ*hxgprt* strain. Parasites expressing P4-ATPase1-BirA-3xHA and HXGPRT were selected by mycophenolic acid (25 μg/mL) and xanthine (50 μg/mL), as reported elsewhere [59]. The crossover-specific PCR, sequencing and immunostaining methods were deployed to verify the clonal transgenic tachyzoites.

For blotting, the *P4-ATPase1-BirA-3xHA* and parental strains were cultured in the standard medium for 24 h (MoI, 2) and then incubated with 150 μM D-biotin (Sigma-Aldrich) for 20 h. A negative control culture without cofactor was also included. Parasites were harvested by syringe release 44 h post-infection, washed 3x with cold PBS (3000 *g*, 4 °C, 5 min) and resuspended in 50 μL of standard lysis buffer (SLB, 10 mM K_2_HPO_4_, 150 mM NaCl, 5 mM EDTA, 5 mM EGTA, pH 7.4; 0.2 % sodium deoxycholate, 1 % Triton X-100) supplemented with protease inhibitor cocktail (Roth). Samples were incubated on ice for 15 min, followed by centrifugation (20,000 *g*,15 min, 4 °C) to obtain the cell-free extract. Proteins were resolved by SDS-PAGE (6 %), blotted and detected by staining with IRdye-streptavidin (800CW, 1:3000). Immunoblotting was performed using rabbit α-*Tg*Hsp90 (1:10,000), mouse α-HA (1:10,000) and corresponding secondary antibodies to assess the protein loading and P4-ATPase1-BirA-3xHA expression, respectively.

#### 4.1.10 Affinity capture of biotinylated proteins

The assay was performed following a standardized protocol based on previously-described reports. Tachyzoites were precultured for 24 h and then cultured in the absence or presence of D-biotin (150 μM) for 20 h. Cells were harvested, washed 3× with cold PBS (1000 *g*, 4 °C) and lysed for 20 min in 1 mL of SLB-containing protease inhibitor cocktail. The supernatant was collected (20,000 *g*, 15 min, 4 °C) and incubated (3 h, 4 °C, 5 g) with 50 μL of streptavidin-coated magnetic beads (pre-washed 5x by SLB). Beads were subjected to serial washing steps by SLB (3x), 1 M KCl (1x), 0.1 M Na_2_CO_3_ (1x), 2 M urea in 10 mM Tris-HCl (pH 8.0, 1x) and then again by SLB (2x) using a magnetic rack (GenScript, USA). Samples were resuspended in 1 mL PBS, and 90 % fraction (v/v) was subjected to pull-down and mass spectrometry. The remaining sample (10 %, v/v) was analyzed by blotting.

### 4.2 Mass spectrometry of biotinylated proteins

#### 4.2.1 Proteolytic digestion

Samples were processed by single-pot solid-phase-enhanced sample preparation (SP3) [61], [62]. In brief, biotinylated proteins were released from the streptavidin-coated beads by incubation for 5 min at 95 °C in an SDS/biotin-containing buffer (1 % (w/v) SDS, 10 mM biotin, 10 mM TRIS, pH 7.5). The eluted proteins were reduced and alkylated by dithiothreitol and iodoacetamide. Subsequently, 2 μL of carboxylate-modified paramagnetic beads were added to samples (Sera-Mag SpeedBeads, GE Healthcare, 0.5 μg solids/μL in water, [61]). After adding acetonitrile to a concentration of 70 % (v/v), samples were allowed to settle at room temperature for 20 min. Beads were washed twice in water with 70 % (v/v) ethanol and then once with acetonitrile. They were resuspended in 50 mM NH_4_HCO_3_ containing trypsin (Promega) at an enzyme-to-protein ratio of 1:25 (w/w) and incubated overnight at 37 °C. After proteolytic digestion, acetonitrile was added to a concentration of 95 % (v/v), followed by incubation at room temperature (20 min). To increase the yield, supernatants derived from this initial peptide-binding step were subjected to the SP3 peptide purification procedure [62]. Samples were washed with acetonitrile. To recover the peptides, paramagnetic beads from the original sample and corresponding supernatants were pooled in 2% (v/v) dimethyl sulfoxide, sonicated for 1 min and then centrifuged (12,500g, 2 min, 4 °C). Supernatants containing tryptic peptides were transferred into a glass vial for mass spectrometry.

#### 4.2.2 Liquid chromatography-mass spectrometry (LC-MS) analysis

Tryptic peptides were separated using an Ultimate 3000 RSLCnano LC system equipped with a PEPMAP100 C18 5 µm 0.3 × 5 mm trap (ThermoFisher Scientific) and an HSS-T3 C18 1.8 µm, 75 µm × 250 mm analytical reversed-phase column (Waters Corporation). Mobile phase A was water containing 0.1 % (v/v) formic acid and 3 % (v/v) DMSO. Peptides were separated running a gradient of 2–35 % mobile phase B (0.1 % (v/v) formic acid, 3 % (v/v) DMSO in acetonitrile) for 40 min at a flow rate of 300 nL/min. The total analysis time was 60 min, including wash and column re-equilibration steps. The column temperature was set to 55 °C. Mass spectrometric analysis of eluting peptides was conducted on an Orbitrap Exploris 480 instrument (ThermoFisher Scientific). The spray voltage was set to 1.8 kV, the funnel RF level to 40, and the heated capillary temperature was kept at 275 °C.

Data were acquired either in data-dependent acquisition (DDA) or in data-independent acquisition (DIA) mode. For DDA, the ten most abundant peptides (Top10) were targeted for fragmentation. Full MS1 resolution was set to 120,000 at *m/z* 200, and the automated gain control (AGC) target to 300 % with a maximum injection time of 50 ms. The mass range was set to *m/z* 350–1500. For MS2 scans, the collection of isolated peptide precursors was limited by an ion target of 1 × 10^5^ (AGC target value of 100 %) and maximum injection times of 25 ms. Fragment ion spectra were acquired at a resolution of 15,000 at *m/z* 200. The intensity threshold was adjusted at 1E4. The isolation window width of the quadrupole was set to *m/z* 1.6*,* and the normalized collision energy was fixed at 30 %. In DIA mode, full MS1 resolution was set to 120,000 at *m/z* 200 and AGC target to 300 %. The mass range was set to *m/z* 345–1250. Fragment ion spectra were acquired with an AGC target value of 1000 %, applying a DIA scheme consisting of 21 windows with variable width and a 0.5 Th overlap. The resolution was set to 30,000, and ion transfer time was determined in “auto mode”. The normalized collision energy was fixed at 27 %. Data were acquired in profile mode using positive polarity.

#### 4.2.3 Data analysis and label-free quantification

The DDA-derived raw data acquired with the Exploris 480 were processed with MaxQuant (v2.0.1) [63], [64] using standard setting and label-free quantification (LFQ) enabled for each parameter group, *i.e.*, *P4-ATPase1-BirA-3xHA* and parental samples (LFQ min ratio count 2, stabilize large LFQ ratios disabled, match-between-runs). Data were searched against the forward and reverse sequences of the *T. gondii* proteome (UniProtKB/TrEMBL, 8450 entries, UP000005641, released November 2021) and a list of common contaminants. For peptide identification, trypsin was set as a protease, allowing for two missed cleavages. Carbamidomethylation was set as a fixed and oxidation of methionine and acetylation of N-termini as variable modifications. Only peptides with a minimum length of 7 amino acids were considered. Peptide and protein false discovery rates (FDR) were set to 1 %. In addition, proteins were identified based on at least two peptides. Statistical analysis was conducted using the Student’s *t*-test, corrected by the Benjamini-Hochberg (BH) method for multiple hypothesis testing (FDR, 0.01). Only proteins over two-fold enrichment compared to the controls were selected.

The DIA-derived raw data acquired with the Exploris 480 were processed using DIA-NN (v1.8.0.1), applying the default parameters for library-free database search. Data were analyzed using a custom-compiled database containing UniProtKB/TrEMBL entries of the *T. gondii* proteome and common contaminants. For peptide identification and *in-silico* library generation, trypsin was set as a protease, allowing for one missed cleavage. Carbamidomethylation was set as a fixed modification, and the maximum number of variable modifications was set to zero. The peptide length ranged from 7 to 30 amino acids. The precursor *m/z* range was set to 300–1800, and the product ion *m/z* range to 200–1800. For quantification, we used the robust LC (high precision) mode. Cross-run normalization was disabled (“*off*”). We applied the in-built algorithm of DIA-NN to automatically optimize MS2 and MS1 mass accuracies and scan window size. Peptide precursor FDRs were controlled below 1 %.

#### 4.2.4 Data presentation and statistics

Unless stated otherwise, all assays shown in this study were done at least three independent times. Figures illustrating images or making of transgenic strains typically show only a representative of three or more biological assays. Graphs and statistical significance were generated using GraphPad Prism (v8). The error bars in graphs signify means with S.E. from multiple assays. The *p*-values were calculated by Student’s *t*-test (* *p* ≤ 0.05; ** *p* ≤ 0.01; *** *p* ≤ 0.001; **** *p* ≤ 0.0001).

## CRediT authorship contribution statement

NG conceived, coordinated and supervised the work; NG and KC designed the study; KC and XH performed experiments and generated data; UD and ST carried out the proteomics study; KC, ÖGE and NG analyzed results and drafted the manuscript. All authors reviewed and approved the work.

## Acknowledgment and Funding

We thank Grit Meusel (Humboldt University, Berlin) for technical assistance throughout this work. Besides, we acknowledge the parasitology community for sharing selected biological reagents. This work was primarily sponsored by two standard research grants (GU1100/4–3) and a Heisenberg program grant (GU1100/16) awarded to NG by the German Research Foundation (DFG). Additional support for this work was bestowed by the DBT – Wellcome Trust (India Alliance) senior fellowship grant to NG (IA/S/19/1/504263). Financial support to KC was endowed through the China Scholarship Council (CSC). Support for proteomic analysis was provided through a priority program SPP2225 “EXIT” sponsored by DFG. The funders had no role in the design of this study, data collection and analysis, or the decision to publish this manuscript.

## Declaration of Competing Interest

The authors declare that they have no known competing financial interests or personal relationships that could have influenced this work.

## Data Availability

All original data relevant to this study are included herein. The nucleotide and protein sequences of *Toxoplasma* P4-ATPase1–5 and Lem1–3 proteins can be accessed through databases. The MS data are deposited to the ProteomeXchange Consortium (http://proteomecentral.proteomexchange.org) *via* the jPOST partner repository [65] with the dataset identifiers PXD034041 (ProteomeXchange) and JPST001600 (jPOST).
