# Supplementary material for: Apically-located P4-ATPase1-Lem1 complex internalizes phosphatidylserine and regulates motility-dependent invasion and egress in Toxoplasma gondii
Source: Comput Struct Biotechnol J. 2023 Feb 18;21:1893–906. doi: 10.1016/j.csbj.2023.02.032 (PMC10015115; doi:10.1016/j.csbj.2023.02.032)
Supplement: Supplementary file 1 — Supplementary material [file mmc1.pdf]

Apically-located P4-ATPase1-Lem1 complex internalizes phosphatidylserine and regulates motility-dependent invasion and egress in *Toxoplasma gondii*

Kai Chen[1], Xiyu Huang[1], Ute Distler[2], Stefan Tenzer[2], Özlem Günay-Esiyok[1], Nishith Gupta[1, 3]

[1]Department of Molecular Parasitology, Faculty of Life Sciences, Humboldt University, Berlin, Germany; [2]Institute of Immunology, University Medical Center of the Johannes-Gutenberg University, Mainz, Germany; [3]Intracellular Parasite Education and Research Labs (iPEARL), Department of Biological Sciences, Birla Institute of Technology and Science, Pilani (BITS-P), Hyderabad, India

SUPPLEMENTARY INFORMATION

Figure S1: *P4-ATPase1 and P4-ATPase2 are not involved in the internalization of PtdEtn by tachyzoites*. **(A-B)** Histograms and graphs showing the distribution of tachyzoites labeled by PI (A) and NBD (B). Labeling was performed as depicted in Figure 2A. To estimate the NBD-PtdEtn uptake, tachyzoites with low PI signal (>60% living cells) were analyzed in the green channel. Curves show one of the three experiments, whereas graphs signify the means with standard error (*ca.* 20000 cells/strain). **(C)** Exemplary images of the specified parasite strains labeled with NBD-conjugated PtdEtn.

Figure S2: *Tachyzoites cannot translocate PtdCho from their extracellular milieu*. **(A-B)** Distribution of tachyzoites stained by PI (A) and NBD-PtdCho (B). For the methodical illustration, refer to Figure 2A. To quantify the lipid uptake, living tachyzoites (low PI, >90% cells) were examined for the green signal. (*ca.* 20000 cells/strain, n = 3 assays, means ± SE). **(C)** Representative fluorescent images of the indicated tachyzoite strains after incubation with NBD-PtdCho.

Figure S3: *Immunofluorescent co-staining of Lem1-mAID-3xHA with ISP1 and IMC3 proteins in tachyzoites*. The transgenic strain was generated as described in Figure 3B. Parasites were cultured without IAA for 24 h and immunostained with α-HA and α-*Tg*ISP1 to ascertain the apical localization of Lem1 (A) or with α-*Tg*IMC3 antibodies to test its expression in daughter cells (B).

Figure S4: *The ΔP4-ATPase1 and Lem1-mAID-3xHA exhibit a normal replication.* Graphs show the proliferation efficiency of the P4-ATPase1 and Lem1 mutants along with their related parental strains. The data comprise the count of tachyzoites multiplying within vacuoles (500-600 vacuoles, n = 3 assays, means ± SE). To minimize the influence of invasion defect on parasite replication, IAA (500 µM), where applicable, was added 4 h post-inoculation.

Table S1: Oligonucleotides used in this study

Table S2: Mass spectrometry datasets following the proximity-dependent biotinylation assay
