# Supplementary figures and images for: Apically-located P4-ATPase1-Lem1 complex internalizes phosphatidylserine and regulates motility-dependent invasion and egress in Toxoplasma gondii
Source: Comput Struct Biotechnol J. 2023 Feb 18;21:1893–906. doi: 10.1016/j.csbj.2023.02.032 (PMC10015115; doi:10.1016/j.csbj.2023.02.032)

# Figure S1

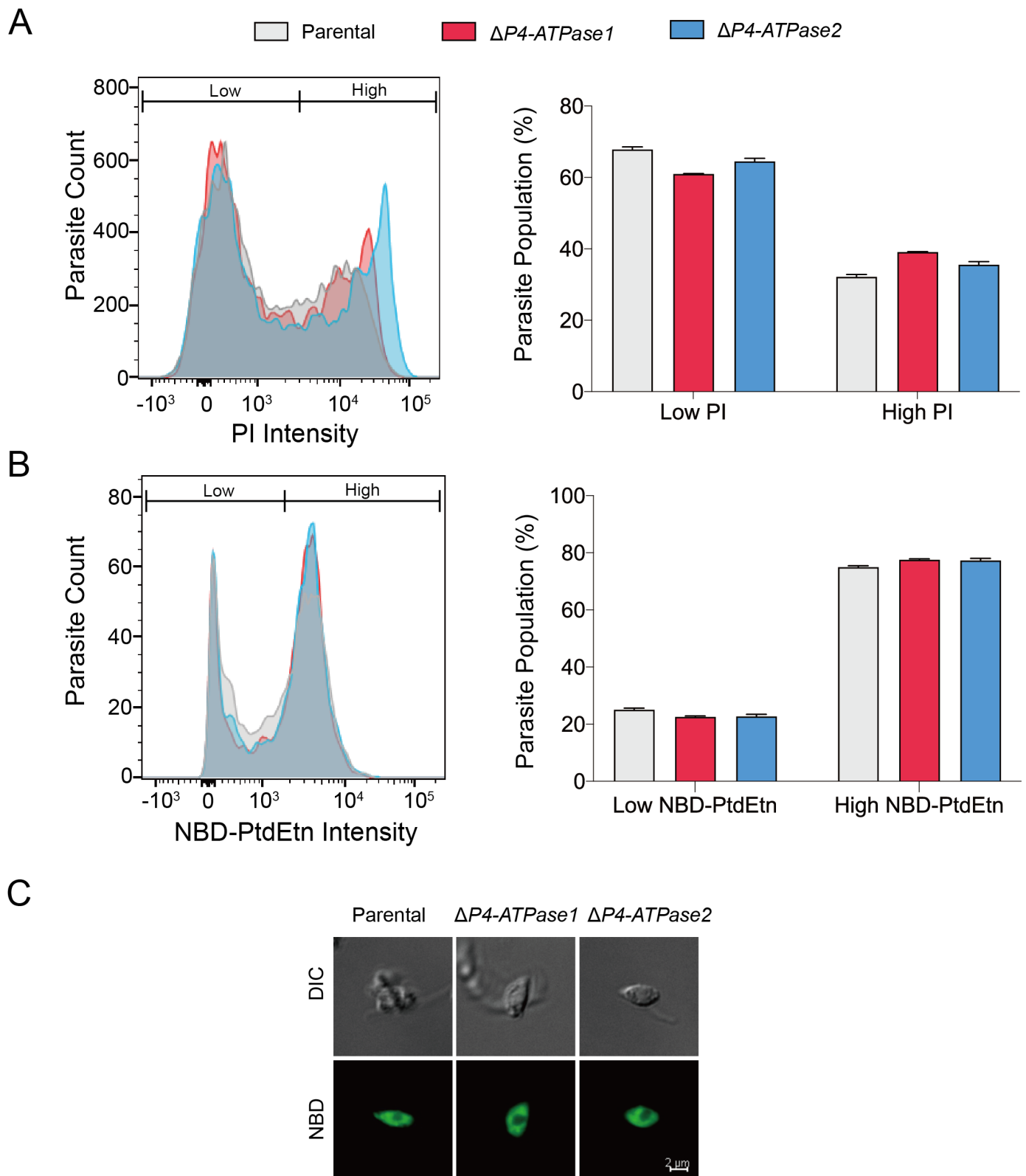

Figure S2

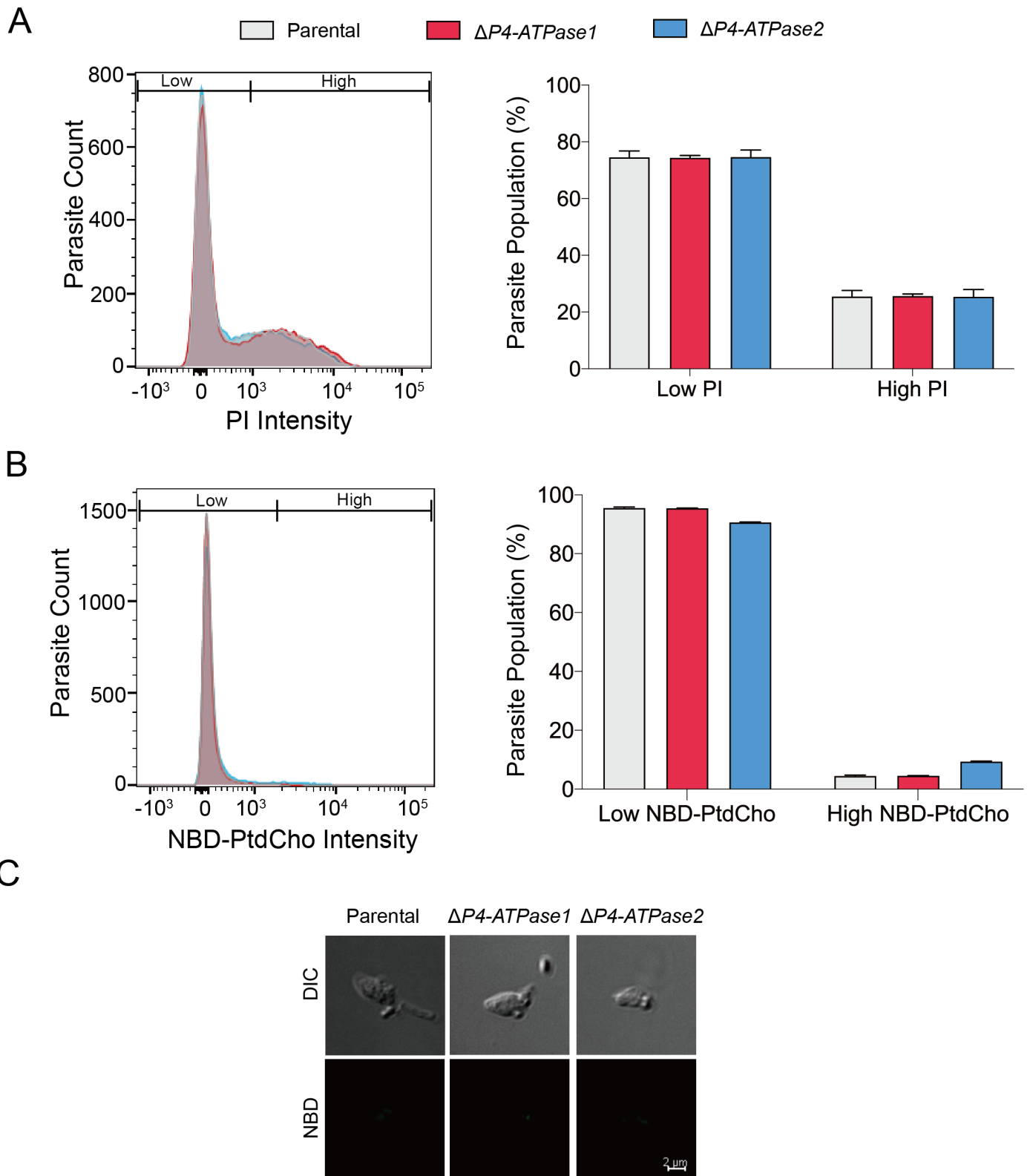

Figure S3

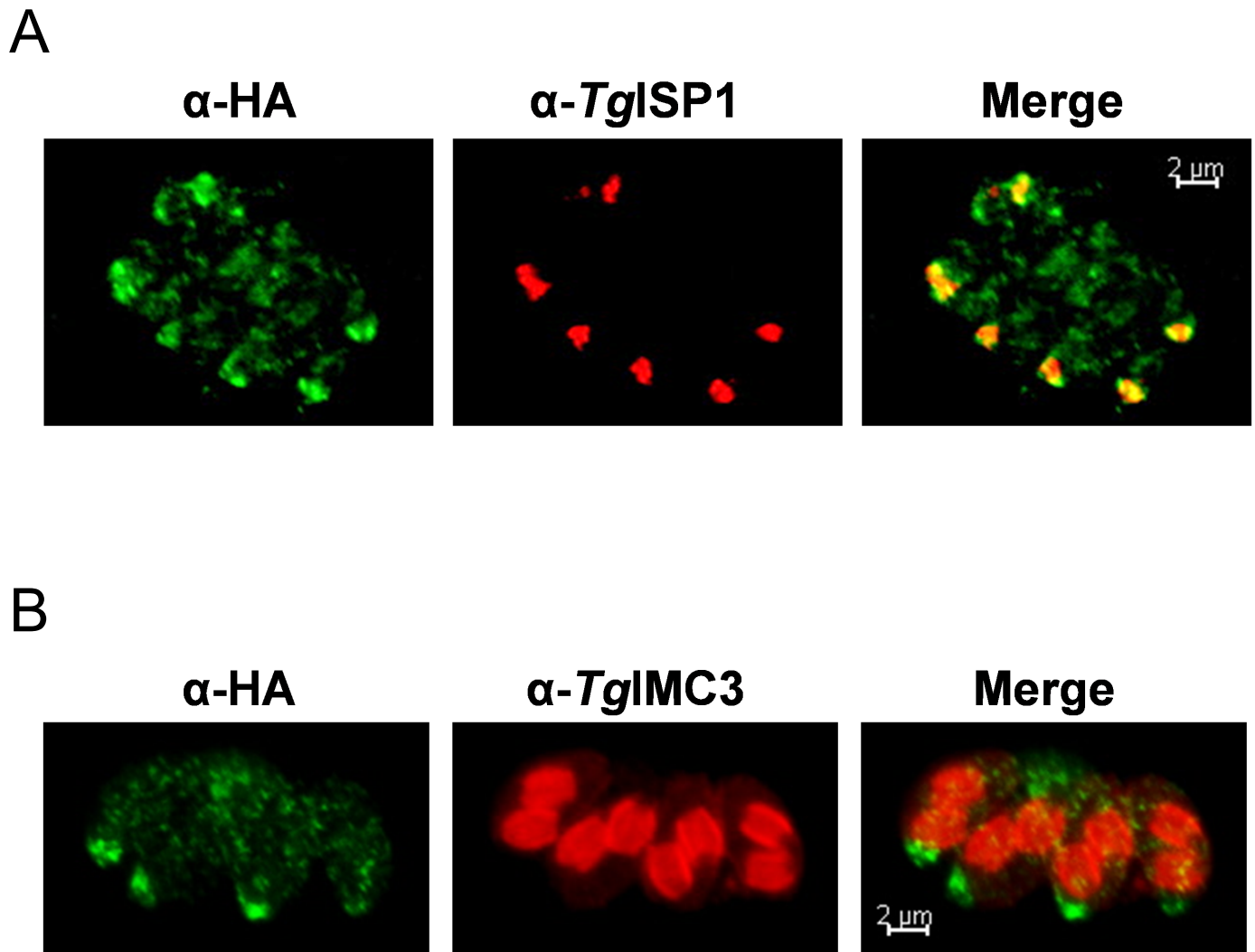

# Figure S4

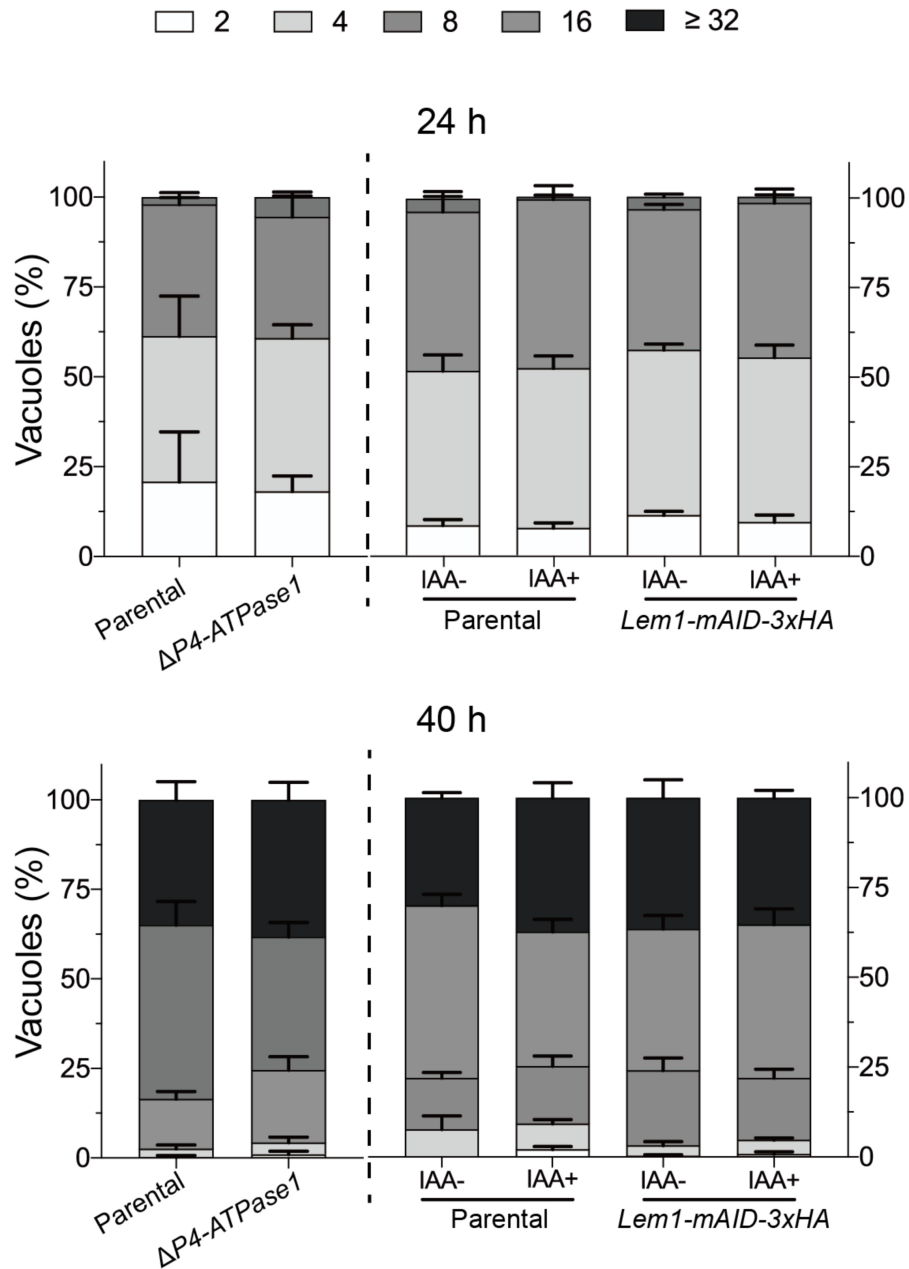

Supplement: Supplementary file 2 — Supplementary material [file mmc2.pdf]
