# Supplementary material for: Apically-located P4-ATPase1-Lem1 complex internalizes phosphatidylserine and regulates motility-dependent invasion and egress in Toxoplasma gondii
Source: Comput Struct Biotechnol J. 2023 Feb 18;21:1893–906. doi: 10.1016/j.csbj.2023.02.032 (PMC10015115; doi:10.1016/j.csbj.2023.02.032)
Supplement: Supplementary file 3 — Supplementary material [file mmc3.pdf]

Table S2: Oligonucleotides, constructs and parasite strains used in this study

**Primer Name**<br>**(restriction site)**	**Nucleotide Sequence**<br>**(*sg*RNA, restriction site or homology arms are underlined)**	**Cloning Vector or Template**<br>**(research objective)**
**Dual CRISPR/Cas9-assisted *Tg*P4-ATPase1/2 knockout in the RHΔ*ku80*Δ*hxgprt* strain**		
*Tg*P4-ATPase1-5'*sg*RNA-F	TTGATCATGGAAGAAGACTGGTTTTAGAGCTAGAAATAGC	***pSAG1-Cas9-U6-sgUPRT***<br>(CRISPR *sg*RNA construction)
*Tg*P4-ATPase1-3'*sg*RNA-F	ATCTGGAACGCTCTGGTCCGGTTTTAGAGCTAGAAATAGC	
*Tg*P4-ATPase2-5'*sg*RNA-F	GACTGTGCTCGTTCTCTCGCGTTTTAGAGCTAGAAATAGC	
*Tg*P4-ATPase2-3'*sg*RNA-F	GTGTGGCGTCCTGCGAGCGGGTTTTAGAGCTAGAAATAGC	
*sg*RNA-Universal-R	AACTTGACATCCCCATTTAC	
3'*sg*RNA-KpnI-F	CGAATTGGGTACCCAAGTAAGCAGAAGCACGCTG	
3'*sg*RNA-XhoI-R	TCGACCTCGAGAATTAACCCTCACTAAAGG	
pUC19-F	GGCGTAATCATGGTCATAGC	***pUC19***<br>(Gibson assembly)
pUC19-R	ATTCGCCCTATAGTGAGTCG	
DHFR-TS-SC-F	CAACCCGCGCAGAAGACATC	
DHFR-TS-SC-R	GGACACGCTGAACTTGTGGC	
*Tg*P4-ATPase1-5'HR-F	CGACTCACTATAGGGCGAATCTCTTCTTCTGTCTGCCTTCTC	***RHΔku80Δhxgprt gDNA template***<br>(Gibson assembly)
*Tg*P4-ATPase1-5'HR-R	GATGTCTTCTGCGCGGGTTGACTCAACTCTCTCCGTCTCTC	
*Tg*P4-ATPase1-3'HR-F	GCCACAAGTTCAGCGTGTCCCCATGCCCTTCCTCCCAAA	
*Tg*P4-ATPase1-3'HR-R	GCTATGACCATGATTACGCCGCTCGGACACTGCTTCCAT	
*Tg*P4-ATPase2-5'HR-F	CGACTCACTATAGGGCGAATCAAGACCTACCACGGAACCT	
*Tg*P4-ATPase2-5'HR-R	GATGTCTTCTGCGCGGGTTGGATTATCGGTCGGACTGTAGC	
*Tg*P4-ATPase2-3'HR-F	GCCACAAGTTCAGCGTGTCCAAGTGAATGTGAGTAAGTGGGA	
*Tg*P4-ATPase2-3'HR-R	GCTATGACCATGATTACGCCACCTACGCTGGACGACTAC	
*Tg*P4-ATPase1-PCR1-F	TGCCTCCACCTGGTTCTTCTCT	***pDrive***<br>(PCR screening to confirm the integration of DHFR-TS cassette)
*Tg*P4-ATPase1-PCR2-R	GCTTGCGAACGCTTCCTGTCT	
*Tg*P4-ATPase1-PCR3-F	CGTTGAAGATGCCTGTGA	
*Tg*P4-ATPase1-PCR3-R	TTGTCGCTGAAGATGTAGG	
*Tg*P4-ATPase2-PCR1-F	ACCGTGGATTGTCGTGGCAGA	
*Tg*P4-ATPase2-PCR2-R	GAAGAATTGCTGCCGCCCTTG	
*Tg*P4-ATPase2-PCR3-F	CTGATGGTGATGGCTCTG	
*Tg*P4-ATPase2-PCR3-R	GCTGCTCCTGTGAAGTAG	
PCR1-R	GCTTCTCCGCCGCAATGTCTT	
PCR2-F	ACACGCATGTCTACACGAACCA	
**CRISPR/Cas9-assisted 3'-insertional tagging (3'IT) of *Tg*Lem1/3 gene with a mAID-3xHA epitope in the RHΔ*ku80*Δ*hxgprt*-Tir1 strain**		
*Tg*Lem1-*sg*RNA-3'IT-F	GCGGCGTGGACAGCGCCTCTGTTTTAGAGCTAGAAATAGC	***pSAG1-Cas9-U6-sgUPRT***<br>(CRISPR *sg*RNA construction)
*Tg*Lem3-*sg*RNA-3'IT-F	AGACAAGTGAAGCTAGAGAGGTTTTAGAGCTAGAAATAGC	
*sg*RNA-Universal-R	AACTTGACATCCCCATTTAC	
*Tg*Lem1-mAID-3xHA-COS-F	AACTGCAGGCGACGCCCTGGAGGATCCTGCTCTAGGCGAGGCTAGCAAGGGCTCGGGC	***pLinker-mAID-3xHA-HXGPRT***<br>(PCR of donor amplicons with 5' and 3' crossover sequences or COS)
*Tg*Lem1-mAID-3xHA-COS-R	TTTCTCTCTTTTTTGGCATTCGTCTGTTTCTTTCCTGCGTATAGGGCGAATTGGAGCTCC	
*Tg*Lem3-mAID-3xHA-COS-F	AGACATTTTGTGGCTGCGAAAAGCTCTTTACGCGGGCAGTGCTAGCAAGGGCTCGGGC	
*Tg*Lem3-mAID-3xHA-COS-R	CAACACTCTTTGGTCGCCGTGCATCGCGCATACATGCCTGATAGGGCGAATTGGAGCTCC	
*Tg*PLem1-mAID-3xHA-Scr-F	CGCTGAACTCCGCCGAAGAA	***pDrive***<br>(PCR screening to confirm the mAID-3xHA tagging of Lem1/3)
*Tg*PLem3- mAID-3xHA-Scr-F	TGGGTGACGAAGAGAGGCTTTG	
*Tg*Lem1/3-mAID-3xHA-Scr-R	CGGCACCACTTCTCGTACTATG	
**CRISPR/Cas9-assisted 3'-insertional tagging (3'IT) of *Tg*P4-ATPase1 gene with a BirA-3xHA epitope in the RHΔ*ku80*Δ*hxgprt* strain**		
*Tg*P4-ATPase1-*sg*RNA-3'IT-F	GACAGTGCAGAAGGATGAGCGTTTTAGAGCTAGAAATAGC	***pSAG1-Cas9-U6-sgUPRT***<br>(CRISPR *sg*RNA construction)
*sg*RNA-Universal-R	AACTTGACATCCCCATTTAC	
*Tg*P4-ATPase1-BirA-3xHA-COS-F	GGAGTGTTACAGGCATCGTGTGCAGCAGTCCAGAGGATTGGCTAGCAAGGGCTCGGGC	***pLinker-BirA-3xHA-HXGPRT***<br>(PCR of donor amplicon)
*Tg*P4-ATPase1-BirA-3xHA-COS-R	TAATTTCGCAGAAGGCTTCCCCGCTCCATGGCTCTTCGCAATAGGGCGAATTGGAGCTCC	
*Tg*P4-ATPase1-BirA-3xHA-Scr-F	CGTAGATCCGCATGACAG	***pDrive***<br>(PCR screening to confirm the BirA-3xHA tagging of P4-ATPase1)
*Tg*P4-ATPase1-BirA-3xHA-Scr-R	CCTGGAGGTACAGGTCATTAG	
